# Local anesthesia type affects cancer detection rate in transrectal ultrasound guided prostate biopsy

**DOI:** 10.1590/S1677-5538.IBJU.2014.0337

**Published:** 2015

**Authors:** Mustafa Zafer Temiz, Engin Kandirali, Aykut Colakerol, Murat Tuken, Atilla Semercioz

**Affiliations:** 1 Department of Urology, Bitlis State Hospital, Besminare, Bitlis, Turkey; 2 Department of Urology, Bagcilar Training and Research Hospital, 6. Sokak, Bagcilar/Istanbul, Turkey

**Keywords:** Neoplasms, Anesthesia, Local, Prostate, Biopsy

## Abstract

**Purpose:**

Studies about the anesthesia techniques during transrectal ultrasound guided prostate biopsy (TRUS-Bx) are usually focused on pain relief. Although patients' tolerance is an important issue in TRUS-Bx, cancer detection rate (CDR) must not be ignored. In this study, we compared the impact of intrarectal lidocaine gel anesthesia (IRLA) and periprostatic nerve blockade (PNB) techniques on CDR.

**Materials and Methods:**

A total of 422 patients underwent 10 core-TRUS-Bx because of elevated serum prostate specific antigen (PSA) level (>2.5ng/mL) and/or suspicious digital rectal examination findings. Patients were divided into two groups according to the applied anesthesia technique: IRLA group and PNB group. Age, serum PSA level, prostate volume, visual analogue scale (VAS) score and CDR were recorded and compared statistically with chi square and unpaired t-tests.

**Results:**

Of the patients 126/422 (29.9%) underwent TRUS-Bx by using IRLA whereas 296/422 (70.1 %) by PNB technique. The mean, age, serum PSA level and prostate volume were similar between the two groups. CDR was 19.8% and 25.4% in IRLA and PNB groups, respectively (p=0.001). The mean VAS score of the PNB group (1.84±0.89) was significantly lower than that for IRLA group (3.62±1.06) (p=0.001). Conclusions: Our results revealed that PNB is superior to IRLA in terms of CDR. Further studies are required to confirm our findings.

## INTRODUCTION

Transrectal ultrasound guided prostate biopsy (TRUS-Bx) is a widely performed procedure in the diagnosis of prostate cancer. Although it is considered a minor and well-tolerated procedure, 65% to 90% of the patients complain about pain (1, 2). Local anesthesia prior to biopsy is a crucial part of TRUS-Bx for pain control. Several methods of local anesthesia for TRUS-Bx are available, including periprostatic nerve blockade, topical rectal administration or intraprostatic injection of local anesthetics (3).

Numerous studies regarding anesthesia techniques compared the efficacy of pain management during TRUS-Bx (4–6). Although patient tolerance is an important issue in TRUS-Bx, CDR must not be ignored. To our knowledge, there is no clinical study which primarily compares the CDR with different anesthesia techniques during TRUS-Bx. Thus, the aim of this study was to determine the impact of intrarectal lidocaine gel anesthesia (IRLA) and periprostatic nerve blockade (PNB) on CDR following TRUS-Bx.

## MATERIAL AND METHODS

Between February 2009 and December 2012, 526 men who underwent TRUS-Bx at our institution were included for this retrospective study. The institutional review board approved the protocol and all participants provided their informed consent for TRUS-Bx prior to the procedure. Indications for biopsy were elevated serum PSA levels (>2.5ng/mL) and/or suspicious digital rectal examination findings.

Exclusion criteria included previous prostate biopsies, lidocaine allergy, hemorrhagic diathesis, recto-anal pathology, diabetes mellitus, neurologic diseases, and inabilities to rate visual analog scale (VAS). Moreover, patients who were diagnosed with high grade prostatic intraepithelial neoplasia (HG-PIN) and/or atipic small acinar proliferation (ASAP) on pathologic evaluation of the initial TRUS-Bx were not included either.

All patients who had sterile urine culture before the procedure received an enema on the morning of the procedure. Oral levofloxacin (500mg daily, for 5 days, started the night before the biopsy) was given. All procedures were performed by an urologist of our clinic. After the patients being positioned on left lateral decubitus, either intrarectal 6mL 2% lidocaine HCl gel (Aqua Touch Jelly; Istem Medical, Turkey) was applied digitally on the anterior anal wall and prostate surface (group IRLA) or PNB was performed with 5mL 1% lidocaine which was bilaterally injected with a 18 Gauge spinal needle (Gallini Medikal Devices, Italy) into the region of the prostatic vascular pedicle on each side (group PNB). The choice of anesthetic methods was completely up to the urologist who performed the procedure. After administration of the local anesthetics, prostate volumes were measured by using the prostate ellipse formula (7) and prostate gland was evaluated sonographically (Pro Focus 2202 color, Prostate Triplane 8818, 4–12 MHz; BK Medical, Denmark). Afterwards, 10 cores systematic TRUS-Bx was performed via 25cm 18 Gauge tru-cut biopsy needle (Gallini Medikal Devices, Italy) and an automatic biopsy gun (Pro-Mag Ultra-Angiotech, Denmark). Each patient was asked to rate the severity of pain during the procedure on a 10cm visual analogue scale (VAS).

All complications such as vasovagal hypotension, hematuria, rectal bleeding, urethrorrhagia, hematospermia, lower urinary tract symptoms (LUTS), fever, and other possible complications during and after the procedure were recorded. Patients were invited for follow-up after 10 days of the procedure.

Patient characteristics, mean VAS score and CDR were compared between the two groups.

Unpaired t-test and chi-square test were used for the statistical analyses. A p value<0.05 was considered statistically significant.

## RESULTS

Of the 526 patients, 422 (80.2%) who met the inclusion criteria were included to the study. The mean age, serum PSA level and prostate volume of patients were 64.5±7.9 years, 58.1±27.7cc and 12.8±17.2ng/mL, respectively. TRUS-Bx was performed with IRLA in 126/422 (29.9%) whereas 296/422 (70.1%) patients received PNB. The characteristics of the patients in IRLA and PNB groups are shown on Table-1.

**Table 1:** Data of the patient characteristics, VAS score and CDR in IRLA and PNB groups.

	IRLA group	PNB group	P value
**N**	126	296	
**AGE (YEARS)**	64.7±7.8	64.4±7.9	0.8^a^
**PSA (NG/ML)**	11.6±14.3	13.4+18.3	0.06^a^
**PROSTATE VOLUME (CC)**	60±26	57±28.4	0.6^a^
**VAS SCORE**	3.62+1.06	1.84±0.89	0.001^a^
**CDR (%)**	19.84 %	25.42 %	0.001^b^

a Student t test

b Chi-square test

**CDR =** Cancer Detection Rate

**VAS =** Visual Analog Scale

There were no statistical differences on digital rectal examination findings. Suspicious examination rates were 14.9% and 15.1% in IRLA and PNB groups, respectively (p=0.8).

The groups were similar in terms of mean age, prostate volume and serum PSA levels (p>0.05 for each). Mean VAS score was statistically lower in IRLA group compared to that for the PNB group (p=0.001). CDR was 25.42% and 19.84% in IRLA and PNB groups, respectively and the difference was statistically significant (p=0.001).

Gleason scores rates were similar between the groups. Gleason score≤6 rates were 46.2% and 41.7% and Gleason score≥7 rates were 53.8% and 58.3% in IRLA and PNB groups, respectively (p=0.1).

There were only minor complications, such as vasovagal hypotension, mild hematuria, rectal bleeding, hematospermia and LUTS all of which were managed conservatively. No significant difference was found between the groups when the complications were taken into consideration (Table-2).

**Table 2:** Comparison of the minor complications after the procedure in IRLA and PNB groups.

	IRLA group	PNB group	P value
**vasovagal hypotension (n/%)**	8/6.3	17/13.4	0.7^a^
**Mild hematuria (n/%)**	62/49.2	149/50.3	0.4^a^
**Rectal Bleeding (n/%)**	17/5.7	46/15.5	0.08^a^
**hematospermia (n/%)**	12/9.5	34/11.4	0.09^a^
**LUTS (n/%)**	7/5.5	18/6	0.7^a^

LUTS = Lower urinary tract symptoms.

a Chi-square test

## DISCUSSION

TRUS-Bx is the standard method for diagnosis of prostate cancer (8). Prostate biopsy has evolved from digitally guided biopsy technique to the standard sextant transrectal ultrasound guided method (9, 10). Although TRUS-Bx is regarded as a safe and minimal invasive technique, patients experience significant discomfort during the procedure (11). Several clinical studies demonstrated that up to 20% of patients report significant pain during TRUS-Bx and they would refuse re-biopsy without analgesia (12).

Thus, many strategies are adopted to reduce pain and enhance patient tolerance during this procedure.

Pain during biopsy is mainly caused by the introduction and manipulation of the ultrasound probe in the anal canal and penetration of the needle into the prostate capsule, which is richly innervated with autonomic sympathetic and parasympathetic fibers (13, 14). Probe manipulations are necessary to obtain samples from different regions of the prostate (e.g. far-lateral and apex) with adequate imaging in TRUS-Bx. The prostatic apex is a common site of cancer detected by traditional biopsy techniques (15). Similarly, some studies found that addition of laterally directed biopsies of the base, mid-gland, and apex resulted in a 14–17% increase in CDR (16, 17). Importantly, when repeat biopsies are considered, assurance that the far-lateral region and the apical region were sampled appears to be essential because disease in these sites is frequently missed during first biopsy (15). Therefore, manipulation of the ultrasound probe is necessary for an effective biopsy procedure in order to enhance CDR which may be associated with increased patient discomfort.

Berger et al. reported a significant reduction in the level of discomfort from prostate biopsy and transrectal probe manipulation after administration of periprostatic lidocaine. The authors stated that the lidocaine depot had a local effect on the autonomic innervation of the rectal wall (18). In addition to this, anesthetic blockade of the capsular sensation fibers with PNB can decrease the level of patient anxiety and makes the examination more tolerable due to decrease in pelvic muscle contraction (19). Therefore, probe manipulation may be easily done with less patient discomfort as a result of decreased pelvic muscle contraction and pain during TRUS-Bx.

In our study, mean VAS score was lower in PNB group which confirms the findings of Berger et al. (18) Furthermore, CDR was also higher in the PNB group. Ameliorated patient comfort during TRUS-Bx via PNB may enable the sonographer manipulate the probe for better visualization of the gland and obtain biopsies from the apex and far lateral parts of the prostate. This advantage of PNB may play a role in improved CDR compared to IRLA.

Application of perianal-intrarectal topical anesthetic creams or gels to reduce probe related pain during TRUS-Bx is a controversial issue. Some studies reported effective pain relief during probe manipulation with perianal-intrarectal gel anesthesia, whereas others did not (20–22). Since rectal mucosa has a rich absorptive capacity, topical anesthetic gel administered intrarectally may spread into the circulation which may decrease its local effects (23, 24). In the present study, VAS score was higher in IRLA group which may be related to decreased CDR. In our opinion the reason is that, limited manipulation of ultrasound probe with IRLA may avoid sampling apical and far lateral zones where prostate cancer can commonly exists (15).

Various variables may influence the cancer detection rates and diagnostic yield of prostate biopsies such as; patient age and race, serum PSA level, prostate volume, biopsy quality and method of biopsy (e.g., random or ultrasound guided etc.), operator skill (e.g., learning curve etc.), location and number of cores, core length (25, 26). In our study, all of these parameters were similar in each group, except the core length which was not evaluated. According to core length analysis obtained with PNB, it is likely that the adequate core lengths may have improved CDR in our study. This issue must be addressed in further studies.

Nowadays in clinical practice, optimum anesthetic method for interventional procedures is general anesthesia (27). According to our findings, general anesthesia may further improve CDR theoretically because of its maximal patient compliance effect. However, we think that routine usage of general anesthesia during TRUS-Bx does not seem possible because of its invasiveness and expensiveness.

Some limitations of our study merit consideration. 1: There is a difference in number of patients between the two groups. The retrospective nature of our study design is responsible for this phenomenon. Further prospective studies may bypass this limitation. 2: Evaluation of the pain score that is especially related to probe manipulation could have strengthened the conclusions of our study. Further studies about this topic must be considered. 3: The zones where prostate cancer was detected should have also been compared. 4: Clinical significance of prostate cancers was not evaluated in this study. We have especially evaluated cancer detection rates. This issue must be evaluated with further studies in order to determine which anesthetic method is superior for diagnosis of clinical significant cancers.

## CONCLUSIONS

Our results revealed that PNB is superior to IRLA in terms of CDR. We concluded that PNB is an useful anesthesia method in TRUS-Bx for effective procedure as well as for pain relief. Further studies are required to confirm our findings.
